# Elevated mid-trimester 4-h postprandial triglycerides for predicting late-onset preeclampsia: a prospective screening study

**DOI:** 10.1186/s12967-022-03261-6

**Published:** 2022-02-08

**Authors:** Qing Liu, Zhihong Zhu, Wen Cai, Liu Yang, ShuangDi Li, Jiarong Zhang

**Affiliations:** 1 Department of Obstetrics and Gynecology, Shanghai ZhongShan Hospital, Fudan University, 180 Fenglin Road, Shanghai, China; 2 Department of Obstetrics and Gynecology, ShangHai General Hospital, Shanghai Jiaotong University, 100 Haining Road, Shanghai, China; 3 Department of Obstetrics and Gynecology, Shanghai First Maternity and Infant Hospital, School of Medicine, Tongji University, 2699 Gaoke West Road, Shanghai, China

**Keywords:** Hypertriglyceridemia, Preeclampsia, OLTT, Prediction, Metabolism

## Abstract

**Background:**

Abnormal maternal lipid concentrations are associated with increased risk of preeclampsia. However, previous studies mainly focused on fasting lipid concentrations, scarce data have been published on the relationship between postprandial triglyceride (TG) concentrations in the second trimester and the risk of preeclampsia. Our aim is to evaluate the potential of triglyceride (TG) concentrations at the time of oral lipid tolerance test (OLTT) measurement in the second trimester to predict preeclampsia and to elucidate the lipid metabolic changes related to these diseases.

**Methods:**

This is a prospective cohort study of Pregnant women at 12–24 weeks of gestation undergone an OLTT in a university affiliated hospital between May 2019 and January 2020. Data were stratified into binaries according to the OLTT results. The receiver operating characteristic (ROC) curve analysis was conducted to determine the optimal cut-off points of TG, HDL-C, LDL-C, sd-LDL, FFA, and BG for predicting preeclampsia.

**Results:**

438 pregnant women were recruited to undergo an OLTT at 12–24 weeks of gestation. Among these, 24 women developed preeclampsia and 414 women remained normotensive. Women who subsequently developed preeclampsia had higher concentrations of 4-h postprandial TG than those who remained normotensive. In the linear logistic regression analyses of potential confounding factors, mid-trimester 4-h postprandial TG concentrations at the time of OLTT measurement were significantly higher in preeclamptic cases than in controls.

**Conclusions:**

Dyslipidemia in the second trimester of pregnancy, particularly postprandial hypertriglyceridemia, appears to be associated with an increased risk of preeclampsia. Mid-trimester 4-h postprandial TG concentration at the time of OLTT measurement may be a potential predictive marker of preeclampsia.

*Trial registration* Data of registration: 2018/10/15**.** Date of initial participant enrollment: 2019/05/01**.** Clinical trial identification number: chiCTR1800018884**.** URL of the registration site: http://www.chictr.org.cn/showproj.aspx?proj=25526**.** Data sharing information: The data including individual participant data, detailed study protocols, statistical analysis plans will be shared upon request to the corresponding author.

**Supplementary Information:**

The online version contains supplementary material available at 10.1186/s12967-022-03261-6.

## Background

Preeclampsia is a multifaceted condition, and its underlying etiologies are still poorly defined. Preeclampsia is associated with high morbidity and mortality; it affects 5%-7% of all pregnant women and is responsible for > 70,000 maternal and 500,000 fetal deaths annually worldwide [1]. It is a leading cause of maternal death, severe maternal morbidity, maternal intensive care admissions, cesarean section, and premature birth. Hypertensive disorders of pregnancy, in particular preeclampsia, are also recognized as a major risk factor for cardiovascular disease later in life for both the woman and her child [2].

The increased risks of preeclampsia are associated with maternal obesity, polycystic ovarian syndrome, and pre-existing and gestational diabetes mellitus (GDM). These are all syndromes of maternal metabolic disturbance and, to varying degrees, abnormal maternal lipid concentrations [3]. According to a recent systematic review, including 29 studies, hypertriglyceridemia preceded the onset of preeclampsia. More specifically, within the meta-analysis of five cohort studies, hypertriglyceridemia during the second trimester was associated with the development of preeclampsia, overall suggesting that hypertriglyceridemia may be involved in the development of preeclampsia [4]. Increasingly, the role of lipids has been recognized as critically important in vascular risk modification, and it is possible, therefore, that lipids in pregnancy has an impact on placental vascular development, which may be critical in the development of preeclampsia [5].

During pregnancy, the lipid concentrations increase gradually, resulting in a physiological hyperlipidemia state. Previous studies mainly focused on fasting lipid concentrations, neglected that we are in a postprandial state in most of the day [6, 7]. Postprandial dyslipidemia can reflect the decreased ability of pregnant women to metabolize and clear lipids, which occurs before fasting dyslipidemia. However, only a few studies have characterized the relationship between postprandial triglyceride (TG) concentrations in the second trimester and the risks of preeclampsia.

The postprandial response to an oral lipid tolerance test (OLTT) is a better metabolic challenge to emulate nutrient overload in comparison to an oral glucose tolerance test (OGTT). OLTT is used for screening dyslipidemia [8]. Although OGTT is widely used in prenatal care, OLTT application is extremely rare. Here, we tested the hypothesis that elevated postprandial TG concentrations are correlated with an increased likelihood of preeclampsia in Chinese women. We aimed to determine whether pregnant women with abnormal OLTT results during the second trimester are at a high risk of developing preeclampsia.

## Materials and methods

### Participants and ethical statement

**Fig. 1:**
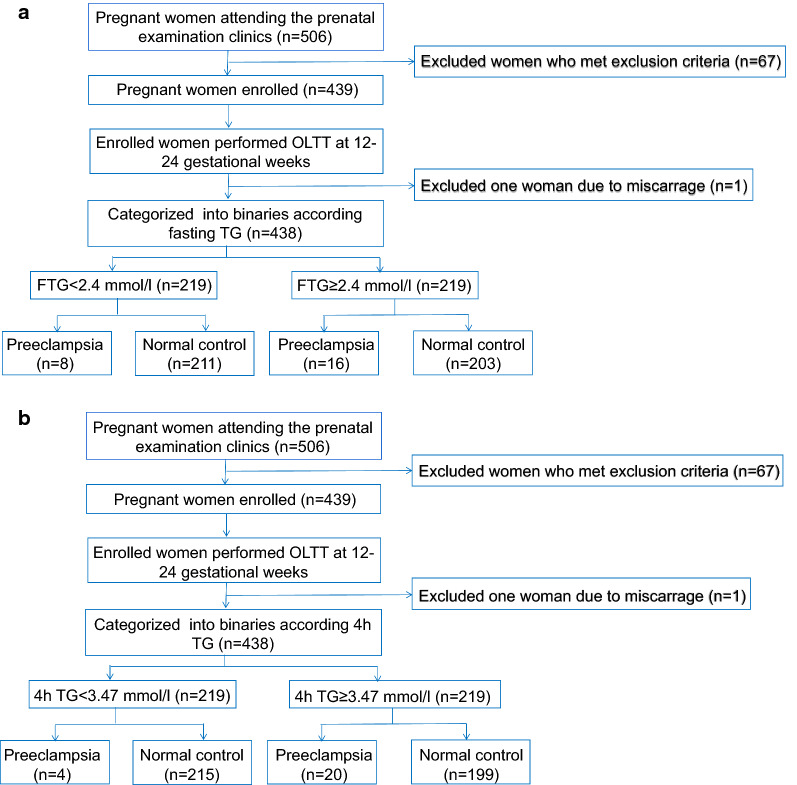
Subject screening and distribution. **A** Subjects distributed by fasting triglyceride. **B** Subjects distributed by postprandial triglyceride

This prospective cohort study enrolled 506 pregnant women who attended the prenatal examination clinics in Shanghai General Hospital between May 2019 and January 2020. In the present analysis, we excluded one woman who had a miscarriage in the second trimester, and 67 women who met the following exclusion criteria: (1) multiple pregnancy; (2) unnatural pregnancy; (3) polycystic ovarian syndrome, thyroid disease, diabetes, heart disease, hypertension, or hyperlipidemia; (4) combined with autoimmune diseases; (5) combined with intrahepatic cholestasis of pregnancy or viral hepatitis; and (6) fasting TG concentrations > 5.6 (mmol/L). The remaining 439 women were included in the final analysis (Fig. 1). These subjects were recruited to undergo an OLTT at 12–24 gestational weeks. A unified standard fat meal referring to the guide was provided for the subjects; the calorie of which was approximately 680 kcal, consisting of a 50-g egg, 30-g butter, 100-g two pieces of toast bread and 250-ml whole milk. The energy proportion of fat, protein, and carbohydrate were 59%, 12% and 29%, respectively [8]. TG, free fat acid (FFA), small dense low-density lipoprotein (sd-LDL) were measured by blood sampling at fasting, and TG was measured at 4 h after the fat meal. Each enrolled pregnant woman underwent a 75-g OGTT at 24–24 weeks of gestation. In addition, fasting lipid concentrations, including serum total cholesterol (TC), TGs, high-density lipoprotein-cholesterol (HDL-C), and low-density lipoprotein-cholesterol (LDL-C) as well as insulin, alanine aminotransferase (ALT), and aspartate aminotransferase (AST) concentrations were measured. Women were diagnosed with GDM if one or more of the following plasma glucose values during the 75-g OGTT at 24–28 gestational weeks were met or exceeded: 0 h, 5.1 mmol/L; 1 h, 10.0 mmol/L; and 2 h, 8.5 mmol/L. Late onset preeclampsia was defined as systolic blood pressure ≥ 140 mmHg and/or diastolic blood pressure ≥ 90 mmHg on at least two occasions 4 h apart developing after 28 weeks of gestation in previously normotensive women. Each patient provided informed consent, and the research was conducted in compliance with the Declaration of Helsinki. This trial has been registered in Chinese Clinical Trial Registry (chiCTR1800018884). The study protocol was approved by the ethics committee of Shanghai General Hospital, Shanghai Jiao Tong University (approval No.: JRS [2018] No.26; approval date: June 20, 2018).

### Grouping

All pregnant women were categorized into the following binaries according to the OLTT results at the mid-trimester: low binary [fasting TG (FTG) < 2.4 mmol/L(median); 4 h TG < 3.47 mmol/L(median)] and high binary (FTG ≥ 2.4 mmol/L; 4 h TG ≥ 3.47 mmol/L) groups. The women were also categorized into the following two groups according to the blood pressure levels and the presence or absence of proteinuria: normal and preeclampsia groups (FTG binary, Fig. 1A; 4 h TG binary, Fig. 1B).

### Data collection

The following maternal characteristics were assessed: age at delivery, height, body weight before pregnancy, blood pressure, and insulin treatment during pregnancy.

### Metabolic measurements

Plasma glucose (PG) concentrations were measured enzymatically. Fasting insulin (FINS) and serum lipid (TC, TG, HDL-C, LDL-C, sd-LDL, and FFA) concentrations were determined by chemiluminescent assays. Hemoglobin A1c was determined by high-performance liquid chromatography. Homeostasis model assessment for β cell function (HOMA-β) and homeostasis model assessment for insulin resistance index (HOMA-IR) were calculated to evaluate β cell function and insulin resistance using the following formulas: HOMA‐β = 20 × FINS(μU/mL) / FPG (mmol/L) − 3.5) and HOMA‐IR = FPG (mmol/L) × FINS (μU/mL) / 22.5.

### Statistical analysis

Data were expressed as the mean ± standard deviation for normally distributed variables and as the median with the interquartile range for skewed data. To determine the differences between the groups, we conducted an analysis of variance or the Kruskal–Wallis test for continuous variables and a chi-square test for categorical variables. Least significant difference tests were used to perform pairwise comparisons between two groups. The receiver operating characteristic (ROC) curve analysis was conducted to determine the optimal cut-off points of TG, HDL-C, LDL-C, sd-LDL, FFA, and BG for predicting preeclampsia. Each optimal cut-off point was assessed by searching for the maximum value of sensitivity + specificity–1 (Youden index). The area under curve (AUC) was calculated to evaluate the predictive powers. Logistic regression analysis was performed to determine whether the elevated postprandial TG concentrations during mid-gestation were independently associated with preeclampsia. Statistical significance was set at p < 0.05. Statistical analyses were performed using SPSS (IBM Corp., Armonk, NY).

## Results

### Demographic and metabolic characteristics of all patients at the time of OLTT measurement

There were significant differences in the metabolic levels during pregnancy between the high and low fasting TG groups. The high fasting TG binary group had a higher pre-pregnancy body mass index (BMI) (22.39 ± 2.11 versus 20.82 ± 1.78 kg/m^2^, *p* < 0.01), higher FPG concentrations (4.71 ± 0.46 versus 4.59 ± 0.35 mmol/L, *p* = 0.03), higher HOMA-IR (1.60 (1.14–2.23) versus 1.23 (1.01–1.66), *p* < 0.01), greater neonatal weight (3318.93 ± 418.02 versus 3260.50 ± 415.58 g, *p* = 0.03), and lower HDL-C concentrations (1.84 ± 0.35 versus 2.05 ± 0.39 mmol/L, *p* < 0.01) than the low fasting TG binary group (Table 1).

**Table 1:** Demographic and metabolic characteristics of all pregnant women at 12–24 gestational weeks who were categorized into binaries according to their fasting TG concentrations at the time of OLTT measurement

Variables	Low binary	High binary	*p* value
	(FTG < 2.4 mmol/L)	(FTG ≥ 2.4 mmol/L)	
	( N = 219)	( N = 219)	
Age (years)	30.95 ± 3.7	30.68 ± 4.03	0.61
Gravidity	2.32 ± 1.12	2.31 ± 1.29	0.95
Parity	0.54 ± 0.34	0.56 ± 0.72	0.91
Prepregnancy BMI (kg/m^2^)	20.82 (19.59–23.15)	22.39 (20.62–24.83)	< 0.01
Systolic Blood Pressure (mmHg)	113.29 ± 14.51	113.9 ± 10.36	0.71
Diastolic Blood Pressure (mmHg)	68.04 ± 8.23	67.48 ± 10.34	0.66
HbA1c (%)	5.00 (4.90–5.20)	5.10 (4.90–5.30)	0.29
FPG (mmol/L)	4.59 ± 0.35	4.71 ± 0.46	0.03
1 h PG (mmol/L)	7.94 ± 1.75	8.28 ± 1.76	0.16
2 h PG (mmol/L)	7.00 ± 1.41	7.11 ± 1.31	0.52
Total Cholesterol (mmol/L)	5.88 (5.45–6.55)	5.90 (5.41–6.78)	0.71
HDL-C (mmol/L)	2.05 ± 0.39	1.84 ± 0.35	< 0.01
LDL-C (mmol/L)	3.14 ± 0.79	3.04 ± 0.97	0.42
HOMA-β (%)	124.21 (86.01–159.49)	122.88 (94.77–171.74)	0.12
HOMA-IR	1.23 (1.01–1.66)	1.60 (1.14–2.23)	< 0.01
Neonatal weight (g)	3260.50 ± 415.58	3318.93 ± 418.02	0.03
Preeclampsia(%)	8 (3.70%)	16 (7.30%)	0.229
Adverse Neonatal Outcomes(%)	52 (23.90%)	60 (27.30%)	0.56
Creatinine	53.16 ± 6.84	54.63 ± 7.02	0.07

Data are expressed as the mean ± standard deviation or median (interquartile range), or n (%). *BMI* body mass index, *FPG* fasting plasma glucose, *HbA1c* hemoglobin A1c, *HDL-C* high-density lipoprotein-cholesterol, *HOMA-IR* homeostasis model assessment for insulin resistance index, *HOMA-β* homeostasis model assessment for β cell function, *LDL-C* low-density lipoprotein-cholesterol, *TG* triglyceride, *1hPG* 1-h plasma glucose, *2hPG* 2-h plasma glucose

Although there was no difference in pre-pregnancy BMI, there were significant differences in the metabolic levels during pregnancy in between the high and low 4-h postprandial TG groups. The high 4-h TG binary group had higher HOMA-IR (1.58 (1.15–2.23) versus 1.23 (0.96–1.71), *p* < 0.01), higher FPG concentrations (4.75 ± 0.47 versus 4.60 ± 0.34 mmol/L, *p* = 0.01), and lower HDL-C concentrations (1.83 ± 0.38 versus 2.07 ± 0.39 mmol/L, *p* < 0.01), and lower LDL-C concentrations (2.95 ± 1.00 versus 3.14 ± 0.76 mmol/L, *p* = 0.04) than the low 4-h TG binary group (Table 2). In addition, the prevalence rates of preeclampsia were 1.83% and 9.09% (p = 0.015) in the two groups (4-h TG < 3.47 mmol/L and 4-h TG ≥ 3.47 mmol/L, respectively) (Table 2).

**Table 2:** Demographic and metabolic characteristics of all pregnant women at 12–24 gestational weeks who were categorized into binaries according to their 4-h postprandial TG concentrations at the time of OLTT measurement

Variables	Low binary	High binary	*p* value
	( 4 h TG < 3.47 mmol/L)	( 4 h TG ≥ 3.47 mmol/L)	
	( N = 219)	( N = 219)	
Age (years)	30.98 ± 3.80	30.58 ± 3.99	0.77
Gravidity	2.39 ± 1.19	2.23 ± 1.22	0.33
Parity	0.56 ± 0.80	0.53 ± 0.71	0.49
Prepregnancy BMI (kg/m^2^)	21.45 (19.81–23.53)	21.78 (20.31–24.45)	0.34
Systolic Blood Pressure (mmHg)	114.93 ± 11.26	113.49 ± 11.05	0.84
Diastolic Blood Pressure (mmHg)	68.17 ± 7.73	68.08 ± 10.60	0.76
HbA1c (%)	5.0 (4.9–5.3)	5.1 (4.9–5.3)	0.1
FPG (mmol/L)	4.60 ± 0.34	4.75 ± 0.47	0.01
1 h PG (mmol/L)	7.87 ± 1.71	8.22 ± 1.87	0.16
2 h PG (mmol/L)	6.70 ± 1.31	7.03 ± 1.43	0.35
Total Cholesterol (mmol/L)	5.98 (5.61–6.68)	5.76 (5.29–6.66)	0.14
HDL-C (mmol/L)	2.07 ± 0.39	1.83 ± 0.38	< 0.01
LDL-C (mmol/L)	3.14 ± 0.76	2.95 ± 1.00	0.04
HOMA-β (%)	124.53 (85.85–164.40)	121.32 (96.58–168.19)	0.29
HOMA-IR	1.23 (0.96–1.71)	1.58 (1.15–2.23)	< 0.01
Neonatal weight (g)	3270.34 ± 428.62	3308.29 ± 407.14	0.08
Preeclampsia(%)	4 (1.80%)	20 (9.10%)	0.015
Adverse Neonatal Outcomes(%)	48 (22.00%)	64 (29.10%)	0.23
Creatinine	53.86 ± 7.84	54.65 ± 6.28	0.07

Data are expressed as the mean ± standard deviation or median (interquartile range), or n (%). *BMI* body mass index, *FPG* fasting plasma glucose, *HbA1c* hemoglobin A1c, *HDL-C* high-density lipoprotein-cholesterol, *HOMA-IR* homeostasis model assessment for insulin resistance index, *HOMA-β* homeostasis model assessment for β cell function, *LDL-C* low-density lipoprotein-cholesterol, *TG* triglyceride, *1hPG* 1-h plasma glucose, *2hPG* 2-h plasma glucose

### Demographic and metabolic characteristics of preeclampsia in the second trimester

Among all enrolled 438 patients, 24 patients developed preeclampsia. Maternal plasma lipid and lipoprotein concentrations are shown in Table 3. Women who subsequently developed preeclampsia had higher plasma glucose and 4-h postprandial TG concentrations and HOMA-IR than the normotensive group. However, it was intriguing that the blood pressure, HbA1c, LDL-C was lower in preeclampsia group compared with the control group.

**Table 3:** Demographic and metabolic characteristics of preeclamptic cases and normotensive controls at 12–24 gestational weeks

Variables	Preeclampsia	Control	*p* value
	( N = 24)	( N = 414)	
	( Mean ± SEM)	( Mean ± SEM)	
Age (years)	30.76 ± 3.89	31.83 ± 4.19	0.35
Gravidity	2.31 ± 1.21	2.23 ± 1.01	0.90
Parity	0.53 ± 0.21	0.54 ± 0.15	0.81
Prepregnancy BMI (kg/m2)	8.07 ± 9.71	8.49 ± 12.06	0.89
Systolic Blood Pressure (mmHg)	112.81 ± 12.24	128.12 ± 10.40	0.01
Diastolic Blood Pressure (mmHg)	67.27 ± 9.18	76.72 ± 7.14	0.01
HbA1c (%)	5.06 ± 0.36	5.24 ± 0.23	0.09
FPG (mmol/L)	5.00 (4.70 ± 5.22)	4.65 (4.37 ± 4.86)	0.001
1 h PG (mmol/L)	9.62 (8.34 ± 10.59)	7.94 (6.73 ± 9.12)	0.002
2 h PG (mmol/L)	7.06 (6.47 ± 7.95)	6.91 (6.10 ± 7.81)	0.51
Total Cholesterol (mmol/L)	5.5 (5.28 ± 6.06)	5.96 (5.48 ± 6.74)	0.245
HDL-C (mmol/L)	1.79 (1.41 ± 2.18)	1.92 (1.66 ± 2.21)	0.364
LDL-C (mmol/L)	2.42 (1.96 ± 3.24)	3.13 (2.50 ± 3.57)	0.044
FFA	0.36 (0.28 ± 0.89)	0.41 (0.28 ± 0.80)	0.95
sd-LDL	40.96 (32.80 ± 55.52)	41.13 (31.71 ± 53.65)	0.72
HOMA-β (%)	121.34 (104.61 ± 133.48)	123.06 (92.38 ± 165.20)	0.606
HOMA-IR	2.25 (1.52 ± 2.86)	1.34 (1.04 ± 1.83)	0.004
FTG	2.81 (2.28 ± 3.30)	2.35 (1.88 ± 3.04)	0.12
4 h TG	4.20 (3.63 ± 5.12)	3.38 (2.44 ± 4.33)	0.037
Neonatal weight (g)	3312.63 ± 405.22	2961.66 ± 541.78	0.05

Data are expressed as the mean ± SEM. *BMI* body mass index, *FFA* fasting free fat acid, *FPG* fasting plasma glucose, *FTG* fasting triglyceride, *HbA1c* hemoglobin A1c, *HDL-C* high-density lipoprotein-cholesterol; *HOMA-IR* homeostasis model assessment for insulin resistance index, *HOMA-β* homeostasis model assessment for β cell function, *LDL-C* low-density lipoprotein-cholesterol, *sd-LDL* fasting small dense low density lipoprotein, *SEM* standard error of the mean, *1 h PG* 1 h plasma glucose, *2 h PG* 2 h plasma glucose, *4 h TG* 4-h triglyceride

### Associations between maternal lipid profile and preeclampsia

Table 4 presents the optimal cut-off points of maternal lipid concentrations in the second trimester for predicting preeclampsia. Four-hour postprandial TG concentrations predicting preeclampsia had the strongest predictive power with the largest AUC [0.680 (95% confidence interval [CI] 0.505–0.854)]. The optimal cut-off points for second-trimester FTG and 4-h postprandial TG in predicting preeclampsia were ≥ 2.93 and 3.92 mmol/L, respectively. Besides, the optimal cut-off points for LDL-C, FFA, sd-LDL, and HDL-C in predicting preeclampsia were ≥ 1.8, 1.46, 29.085 and ≤ 2.42 mmol/L, respectively. Moreover, only high 4-h postprandial TG in the second trimester was significantly associated with the morbidity of preeclampsia (*p* = 0.045).

**Table 4:** Optimal cut-off points of maternal lipids in the second trimester for predicting preeclampsia

	AUC (95% CI)	Sensitivity (%)	Specificity (%)	Youden index	Cut-off point	*p* value
FTG	0.637 (0.471–0.803)	0.545	0.729	0.275	2.93	0.126
4 h TG	0.680 (0.505–0.854)	0.727	0.65	0.378	3.92	0.045
HDL-C	0.419 (0.221–0.616)	0.182	0.897	0.078	2.42	0.221
LDL-C	0.32 (0.154–0.485)	1	0.049	0.049	1.8	0.154
FFA	0.511 (0.319–0.703)	0.182	0.99	0.172	1.46	0.903
sd-LDL	0.532 (0.380–0.685)	1	0.202	0.202	29.085	0.717

*AUC* area under curve, *CI* confidence interval, *FFA* fasting free fat acid, *FTG* fasting triglyceride, *HDL-C* high-density lipoprotein-cholesterol, *LDL-C* low-density lipoprotein-cholesterol, *sd-LDL* fasting small dense low density lipoprotein, *4 h TG* 4-h triglyceride

### 4-h postprandial TG concentrations as a predictive biomarker for the development of preeclampsia

To determine whether the elevated 4-h postprandial TG concentrations during the second trimester of pregnancy was a predictive biomarker for preeclampsia, the odds ratios (ORs) for preeclampsia in women with different TG concentrations during the second trimester were calculated. First, compared to the low 4-h TG binary group at 12–24 gestational weeks, the crude OR for preeclampsia was 1.471 (95% CI 1.049–2.062, *p* = 0.025) for the high TG binary group, and the adjusted OR was 5.561 (95% CI 1.189–26.006, *p* = 0.029) when considering for age, gravidity, parity, pre-pregnancy BMI, and TC concentrations (Table 5). In addition to the elevated 4-h TG concentrations, we also found that an abnormal OGTT result (OR: 3.869, 95% CI: 1.179–12.697, *p* = 0.026) was a risk factor for preeclampsia. However, no association was found between other factors and preeclampsia.

**Table 5:** Factors associated with preeclampsia in the logistic regression analysis

	OR (%95CI)	*p* value
OGTT		0.026
Normal	Reference	
High	3.869 (1.179–12.697)	
FTG		0.238
Low	Reference	
High	2.099 (0.613–7.187)	
4hTG		0.029
Low	Reference	
High	5.561 (1.189–26.006)	
Age (years)	1.021 (0.848–1.230)	0.826
Gravidity	1.025 (0.561–1.874)	0.935
Parity	0.985 (0.814–1.193)	0.878
Prepregnancy BMI (kg/m^2^)	1.014 (0.948–1.084)	0.681
Total Cholesterol (mmol/L)	0.971 (0.411–2.291)	0.946
HDL-C (mmol/L)	0.437(0.078–2.442)	0.437
LDL-C (mmol/L)	0.505(0.243–1.051)	0.068

*BMI* body mass index, *95%CI* 95% confidence intervals, *FTG* fasting triglyceride, *HDL-C* high-density lipoprotein-cholesterol, *LDL-C* low-density lipoprotein-cholesterol, *OGTT* oral glucose tolerance test, *OR* odds ratio, *4 h TG* 4-h triglyceride

## Discussion

Early identification of women at a high risk of preeclampsia might enable potential prophylactic treatment to reduce or avoid the onset of symptoms [9, 10]. Preeclampsia is more common and has a lower detection rate^11^. Predictive models for preeclampsia have employed a combination of maternal characteristics as well as biochemical and biophysical markers before 14 weeks of gestation to predict the syndrome at 30–60% sensitivity [10–14].

Our findings are consistent with the results of the few available prospective cohort studies on maternal non-fasting plasma lipid and lipoprotein concentrations in preeclamptic and normotensive pregnancies [15, 16]. We observed an association between maternal dyslipidemia in the second trimester, particularly 4-h postprandial hypertriglyceridemia, and the subsequent risk of preeclampsia. We evaluated both the FTG and 4-h postprandial TG concentrations at the time of OLTT measurement to show that 4-h postprandial TG concentrations might be an independent predictive biomarker for preeclampsia, which might be more meaningful compared to the FTG concentrations (Table 5). This is in line with the results of a comprehensive study [15] showing the associations between non-fasting plasma TG at 18 weeks of gestation and the risk of preeclampsia. In another prospective cohort study [16], a linear increase in preeclampsia risk was observed with increasing non-fasting plasma TG concentrations before 16 weeks’ gestation. However, fasting TG concentrations in the second trimester were not associated with the development of preeclampsia, which was inconsistent with Oya’s cohort study findings (OR: 1.1 (1.1–1.2), *p* = 0.004) [17]. The reasons on why 4-h postprandial TG concentrations might be more meaningful compared to the FTG concentrations for predicting preeclampsia remain unclear. However, it can be seen in our research that high fasting TG was more associated with pre-pregnancy BMI, while postprandial was more related to insulin resistance, and insulin resistance exists in preeclampsia patients (Tables 2 and 3). Moreover, it is showed postprandial TG were associated with incident cardiovascular events while fasting TG showed little independent relationship [18].

Although, the temporality, wherein 4-h postprandial hypertriglyceridemia clearly precedes the onset of preeclampsia, leads us to generate the hypothesis that we may be able to change the natural history of the disease if we intervene early by lowering the postprandial TG concentrations. Maternal lipids offer a potential therapeutic target, and it is worth noting that the current treatments for diabetes in pregnancy, including dietary and lifestyle changes as well as pharmacotherapy, all alter not only maternal glucose but also maternal lipids [3, 18, 19]. Before such an intervention, it would be important to define the normal postprandial triglyceride concentrations in pregnancy and correctly identify women with lipid metabolic disturbances who could benefit most from this therapy.

Women with elevated lipid concentrations are more likely to have preexisting endothelial dysfunction that worsens as a result of the physiological burden of pregnancy; this condition may be further exacerbated by increased maternal vascular inflammation [20]. This was supported with evidence from a murine model, which suggested that, in pregnancy, excessive hyperlipidemia may lead to an accentuated inflammatory response, oxidative stress, and subsequent dysfunction of cerebral arteries, compromising cerebral percussion and contributing to preeclampsia-related neurological complications [21].

The sensitivity of the prediction of preeclampsia using 4-h postprandial TG concentration at the time of OLTT measurement in this study (72.7%) was markedly higher than that of previously published prediction of preeclampsia based on clinical markers such as mean arterial pressure, maternal age, and uterine artery pulsatility index in a cohort of women at 11 + 0 to 13 + 6 gestational weeks (38.5%) [22] and in another nulliparous cohort (37%) [23]. However, the sensitivity at 10% false positive rate of prediction of preeclampsia is low (18.2%).

The link between postprandial hypertriglyceridemia and preeclampsia was still unclear. However, renin-angiotensin system (RAS) may play a role in this process. RAS has been recognized as a ubiquitous system for homeostasis and pathologies. The perturbation of RAS can cause hypertension and congestive heart failure, obesity, neural diseases, diabetes, and hepatic fibrosis [24, 25]. Angiotensin II promotes adipocyte growth and differentiation, which fuel obesity. The high adipose mass in turn leads to high blood lipid level [26]. RAS can also be attributed as on cause of insulin resistance, which involved in the development of type 2 diabetes [27]. Obesity and gestational diabetes are predisposing factors of preeclampsia. It has been showed that abnormal OGTT, FTG and 4hTG results can be risk factors associated with preeclampsia in our study. In pregnancy, the activity of RAS gradually increased and reach 3-to-sevenfold compared to initial values at the end of gestation. Thus, it is plausible that the perturbation of RAS may act as one key regulator of both metabolic disorders including dyslipidemia, glucose intolerance and preeclampsia. Our further research will be focused on the possible mechanism of RAS in preeclampsia.

It has been reported gestational glucose intolerance can be classified into two subgroups as insulin resistant group and insulin sensitive group, which are at differential risk for adverse outcomes [28]. Similarly, from our research, it can be postulated that preeclampsia may be stratified into two subgroups: normal blood lipid group and dyslipidemia group. These two physiologic subtypes may provide opportunities for a more personalized approach to prevent or treat preeclampsia. For example, hypolipidemic drugs such as statins may reduce the rate of the onset of preeclampsia or lower its severity. Lifestyle regulation may also lower blood lipids and prevent preeclampsia. It is of great interest for us to focus on these aspects in future works.

The major strength of our study was its prospective design with complete follow-up of 438 women with OLTT measurement. This study design more accurately reflects the predictive power of 4-h postprandial TG compared to a case–control design. Our high participation (86.6%) and follow-up (99.5%) rates minimized the likelihood of selection bias, which may affect the reported results. Second, the fasting and 4-h postprandial blood lipid concentrations obtained after a unified fat meal in the second trimester suggested that the disordered plasma lipid concentrations precede preeclampsia. The unified standard fat meal and blood sampling collection time can avoid lipid data deviation. Third, the exclusion of pregnant women with high risk factors for metabolic syndrome made the results more convincing. Fourth, compared to the fetus in the first trimester, the fetus in the second trimester grows rapidly and needs more energy. The mother gradually switches to a catabolic condition resulting in an increased breakdown of lipid. Therefore, detecting postprandial lipid concentration changes (the ability of lipid metabolism) in the second trimester is more sensitive. Finally, we used the logistic regression to adjust for a number of confounders to obtain a conclusion.

Several important limitations must be considered when interpreting the results of our study. First, our study included a limited number of cases, which is difficult to overcome given the low incidence of these disorders. Our relatively small number of patients with preeclampsia hindered us from making inferences from some of our analyses. Further studies involving multiple centers and a larger number of patients are necessary in order to validate our findings. In addition, although we compared many potential confounders, we cannot exclude the possibility of the other confounding factors from unmeasured covariates.

## Conclusions

In conclusion, the results of our study served to highlight the importance of 4-h postprandial TG at the time of OLTT measurement in the second trimester as a possible predictive marker of preeclampsia. Moreover, dyslipidemia, particularly 4-h postprandial hypertriglyceridemia, may be of etiologic and pathophysiologic importance in women who developed preeclampsia. Our data may contribute to the development and evaluation of behavioral and medical interventions aimed at reducing the occurrence of these disorders. On the other hand, preeclampsia appears to be a multi-etiological syndrome with heterogeneous biologic pathways.

## Supplementary Information

**Additional file 1: Table S1.** Demographic and metabolic characteristics of all pregnant women at 12-24 gestational weeks who were categorized into binaries according to their fasting TG concentrations at the time of OLTT measurement (GDM cases removed). **Table S2.** Demographic and metabolic characteristics of all pregnant women at 12-24 gestational weeks who were categorized into binaries according to their 4-h postprandial TG concentrations at the time of OLTT measurement (GDM cases removed). **Figure S1.** ROC curve for FTG and 4h-TG to predict preeclampsia (AUC for FTG 0.637, for 4h TG 0.680).

## Data Availability

Data and material can be achieved by email to the corresponding author (E-mail address: maggie1974@126.com).
