# Supplementary material for: Elevated mid-trimester 4-h postprandial triglycerides for predicting late-onset preeclampsia: a prospective screening study
Source: J Transl Med. 2022 Feb 8;20:81. doi: 10.1186/s12967-022-03261-6 (PMC8822777; doi:10.1186/s12967-022-03261-6)
Supplement: Supplementary file 1 — Additional file 1: Table S1. Demographic and metabolic characteristics of all pregnant women at 12-24 gestational weeks who were categorized into binaries according to their fasting TG concentrations at the time of OLTT measurement (GDM cases removed). Table S2. Demographic and metabolic characteristics of all pregnant women at 12-24 gestational weeks who were categorized into binaries according to their 4-h postprandial TG concentrations at the time of OLTT measurement (GDM cases removed). Figure S1. ROC curve for FTG and 4h-TG to predict preeclampsia (AUC for FTG 0.637, for 4h TG 0.680). [file 12967_2022_3261_MOESM1_ESM.docx]

additional files:

**Table S1.** Demographic and metabolic characteristics of all pregnant women at 12-24 gestational weeks who were categorized into binaries according to their fasting TG concentrations at the time of OLTT measurement (GDM cases removed)

| Variables | Low binary | High binary | *p* value |
| --- | --- | --- | --- |
|  | ( FTG<2.28 mmol/L ) | ( FTG≥2.28 mmol/L ) |  |
|  | ( N=148 ) | ( N=166 ) |  |
| Age (years) | 30.78±4.13 | 30.39±3.91 | 0.548 |
| Gravidity | 2.27±1.10 | 2.35±1.34 | 0.21 |
| Parity | 3.21±2.18 | 0.51±0.74 | 0.06 |
| Prepregnancy BMI (kg/m^2^) | 20.70(19.53-22.65) | 21.71(20.20-24.28) | 0.02 |
| Systolic Blood Pressure (mmHg) | 113.78±14.86 | 112.41±10.13 | 0.499 |
| Diastolic Blood Pressure (mmHg) | 67.98±8.20 | 65.93±10.05 | 0.169 |
| HbA1c (%) | 4.60(4.50-4.80) | 4.70(4.50-4.90) | 0.119 |
| FPG (mmol/L) | 4.49±0.31 | 4.57±0.32 | 0.127 |
| 1h PG (mmol/L) | 7.31±1.27 | 7.56±1.30 | 0.211 |
| 2h PG (mmol/L) | 6.55±0.98 | 6.65±1.05 | 0.529 |
| Total Cholesterol (mmol/L) | 5.97±0.88 | 6.11±1.04 | 0.386 |
| HDL-C (mmol/L) | 2.02±0.39 | 1.89±0.34 | 0.03 |
| LDL-C (mmol/L) | 3.12±0.84 | 3.05±0.93 | 0.615 |
| HOMA-IR | 1.18(1.01-1.52) | 1.44(1.16-1.95) | <0.001 |
| Neonatal weight (g) | 3252.29±413.22 | 3375.18±379.41 | 0.054 |
| Preeclampsia | 2(1.35%) | 8(4.82%) | 0.371 |
| Adverse Neonatal Outcomes  Creatinine | 28(18.92%)  54.25±6.59 | 42(25.31%)  56.34±6.88 | 0.443  0.07 |

Data are expressed as the mean ± standard deviation or median (interquartile range), or n (%).BMI: body mass index; FPG: fasting plasma glucose; HbA1c: hemoglobin A1c; HDL-C: high-density lipoprotein-cholesterol; HOMA-IR: homeostasis model assessment for insulin resistance index; LDL-C: low-density lipoprotein-cholesterol; TG: triglyceride; 1hPG: 1-h plasma glucose; 2hPG: 2-h plasma glucose

**Table S2.** Demographic and metabolic characteristics of all pregnant women at 12-24 gestational weeks who were categorized into binaries according to their 4-h postprandial TG concentrations at the time of OLTT measurement (GDM cases removed)

| Variables | Low binary | High binary | *p* value |
| --- | --- | --- | --- |
|  | ( 4h TG<3.36 mmol/L ) | ( 4hTG≥3.36 mmol/L ) |  |
|  | ( N=154 ) | ( N=160 ) |  |
| Age (years) | 30.70±4.12 | 30.46±3.91 | 0.71 |
| Gravidity | 2.33±1.08 | 2.29±1.36 | 0.847 |
| Parity | 3.09±2.41 | 0.51±0.76 | 0.846 |
| Prepregnancy BMI (kg/m^2^) | 21.19(19.69-23.34) | 21.36(19.97-23.73) | 0.055 |
| Systolic Blood Pressure (mmHg) | 114.13±14.41 | 112.01±10.48 | 0.297 |
| Diastolic Blood Pressure (mmHg) | 67.63±8.73 | 66.02±9.73 | 0.337 |
| HbA1c (%) | 4.60(4.50-4.90) | 4.70(4.50-4.80) | 0.066 |
| FPG (mmol/L) | 4.48±0.31 | 4.58±0.32 | 0.044 |
| 1h PG (mmol/L) | 7.36±1.24 | 7.52±1.34 | 0.431 |
| 2h PG (mmol/L) | 6.59±0.99 | 6.62±1.05 | 0.853 |
| Total Cholesterol (mmol/L) | 6.04±0.81 | 6.06±1.11 | 0.929 |
| HDL-C (mmol/L) | 2.03±0.36 | 1.88±0.36 | 0.01 |
| LDL-C (mmol/L) | 3.17±0.79 | 3.01±0.96 | 0.268 |
| HOMA-IR | 1.20(0.97-1.69) | 1.39(1.15-1.93) | <0.001 |
| Neonatal weight (g) | 3253.44±424.91 | 3378.68±364.97 | 0.049 |
| Preeclampsia | 2(1.29%) | 8(5.00%) | 0.367 |
| Adverse Neonatal Outcomes  Creatinine | 32(20.78%)  54.30±7.60 | 38(23.75%)  56.38±5.71 | 0.704  0.07 |

Data are expressed as the mean ± standard deviation or median (interquartile range), or n (%).BMI: body mass index; FPG: fasting plasma glucose; HbA1c: hemoglobin A1c; HDL-C: high-density lipoprotein-cholesterol; HOMA-IR: homeostasis model assessment for insulin resistance index; LDL-C: low-density lipoprotein-cholesterol; TG: triglyceride; 1hPG: 1-h plasma glucose; 2hPG: 2-h plasma glucose

Figure S1


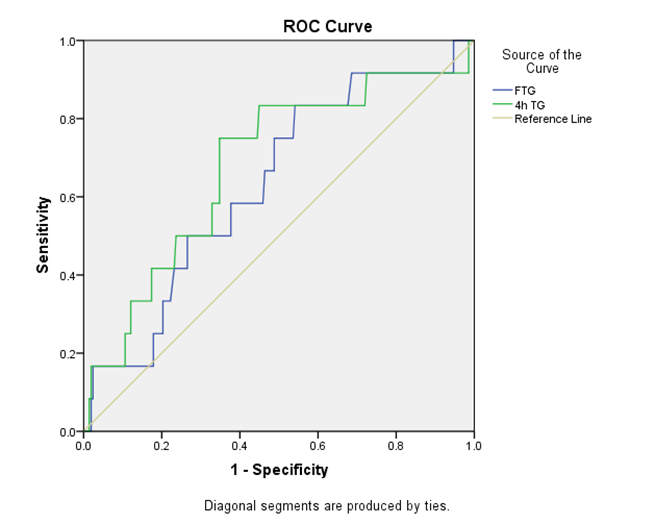


**Figure S1.** ROC curve for FTG and 4h-TG to predict preeclampsia (AUC for FTG 0.637, for 4h TG 0.680)
